# A new Simplified Method of Selective Exposure of Hepatic Pedicles for Controlled Hepatectomies

**DOI:** 10.1155/1989/28161

**Published:** 1989

**Authors:** E. I. Galperin, S. R. Karagiulian

**Affiliations:** Department of Hepatic and Bile Duct Surgery 1st Moscow Medical Institute Hospital No. 7 Kolomensky Pr. 4 Moscow 115487 Russia

## Abstract

Our experience of 90 hepatectomies (HE) and examinations of 64 cadaver livers resulted in the elaboration of a simplified technique for the exposure of hepatic pedicles (HP) and the rapid selective ligation without significant normothermal ischemia of the retained parts of the liver. The method comprises 4 consecutive steps: 1) a superficial T-shaped incision of Glisson's capsule at the site of HP projection on the liver's inferior surface, 2) introduction of the surgeon's forefinger into the liver parenchyma, controlled by clamping the hepatoduodenal ligament, the fingertip finding a tubular structure well distinguished by its smooth elastic surface from the friable parenchyma and bending the finger to hook the pedicle, 3) drawing the hooked pedicle downwards through the slit in the capsule and temporarily clamping it, while releasing the hepatoduodenal ligament so as to restore blood supply to the retained parts of the liver, 4) checking for correct ligature position on the HP before its final ligation by matching the actual ischemic area with the intended line of resection and moving the clamp proximally or distally along the exposed pedicle for the release or clamping of lateral branches as necessary. Whereupon resection can be performed by any of the known methods.

This method has been used in 8 major HE, allowing to reduce intraoperative blood loss from 2200±247 ml to 1000±225 ml and reducing general liver ischemia from 10 minutes and more to 2–3 minutes.


*HPB Surgery* 1989, Vol. 1, pp. 119–130
Reprints available directly from the publisher
Photocopying permitted by license only

© 1989 Harwood Academic Publishers GmbH
Printed in Great Britain

### A NEW SIMPLIFIED METHOD OF SELECTIVE EXPOSURE OF HEPATIC PEDICLES FOR CONTROLLED HEPATECTOMIES

E.I. GALPERIN* and S.R. KARAGIULIAN

*Department of Hepatic and Bile Duct Surgery, 1st Moscow Medical Institute, Hospital No. 7, Kolomensky Pr. 4, 115487 Moscow, USSR*

*(Received 28 July 1988)*

Our experience of 90 hepatectomies (HE) and examinations of 64 cadaver livers resulted in the elaboration of a simplified technique for the exposure of hepatic pedicles (HP) and the rapid selective ligation without significant normothermal ischemia of the retained parts of the liver. The method comprises 4 consecutive steps: 1) a superficial T-shaped incision of Glisson's capsule at the site of HP projection on the liver's inferior surface, 2) introduction of the surgeon's forefinger into the liver parenchyma, controlled by clamping the hepatoduodenal ligament, the fingertip finding a tubular structure well distinguished by its smooth elastic surface from the friable parenchyma and bending the finger to hook the pedicle, 3) drawing the hooked pedicle downwards through the slit in the capsule and temporarily clamping it, while releasing the hepatoduodenal ligament so as to restore blood supply to the retained parts of the liver, 4) checking for correct ligature position on the HP before its final ligation by matching the actual ischemic area with the intended line of resection and moving the clamp proximally or distally along the exposed pedicle for the release or clamping of lateral branches as necessary. Whereupon resection can be performed by any of the known methods.

This method has been used in 8 major HE, allowing to reduce intraoperative blood loss from 2200±247 ml to 1000±225 ml and reducing general liver ischemia from 10 minutes and more to 2–3 minutes.

KEY WORDS: Liver, hepatectomy, hepatic pedicles, access to pedicles

An accurate and least traumatic approach to a hepatic pedicle (HP) in the portion of the liver being removed, is a crucial element of anatomical resection.[6,7,19]

Existing methods of HP exposure presuppose access to them through the parenchymal mass along avascular fissures by the finger fracture technique, or their dissection in the hilus of the liver.[3,7,19] Both these methods entail considerable intraoperative loss of blood.

Our experience of 90 hepatectomies (HE) performed between 1972 and 1987 suggested the idea of drawing an HP out into a small incision on the inferior surface of the liver obviating its careful dissection.[6,7] This idea is based on well-known facts: the lobar and segmental HP are lodged not farther than 2–2.5 cm from the inferior surface of the liver and have a topographical projection onto that surface:[3,13] owing to the epifascial sheath enfolding them, they may be palpably distinguished by their consistency from the surrounding parenchyma, and are sufficiently strong and elastic.[22] The investigation was basically aimed at the simplification of the operation so as to reduce its traumatic effect and bring it within reach of a greater number of surgeons.

* Address correspondence to: E.I. Galperin.

### MATERIALS AND METHODS

Sixty anatomical HE were performed according to the elaborated technique on human cadavers whose cause of death was not associated with hepatobiliary diseases; this method was subsequently used clinically when performing HE in 8 patients.

The most probable location of the HP belonging to the portion of the liver (lobe, sector or segment*) to be removed was determined and projected onto the liver's undersurface relative to its least variable landmarks (gallbladder fossa, porta hepatis and round ligament), whereupon, after clamping the hepatoduodenal ligament (HDL), a 3–3.5 cm long superficial T-shaped incision of the liver capsule was made (Figure 1). Then the operating surgeon introduces his right forefinger upwards into the incision, tunneling the parenchyma until the finger tip finds, at about 1.5–2 cm from the surface, a dense elastic tubular structure which more often than not proves to be the HP being sought, hooks it by bending the finger and draws it out onto the undersurface of the liver (Figure 2 a, b, c) whereupon the exposed HP is clamped and the HDL clamp is released. The borderline between the different coloration of the liver appearing upon HP clamping and HDL release allows to correct and, if necessary move the clamp along the HP (Figures 3 a, b; 4 a, b) before its final ligation so that the line of ischemia coincides

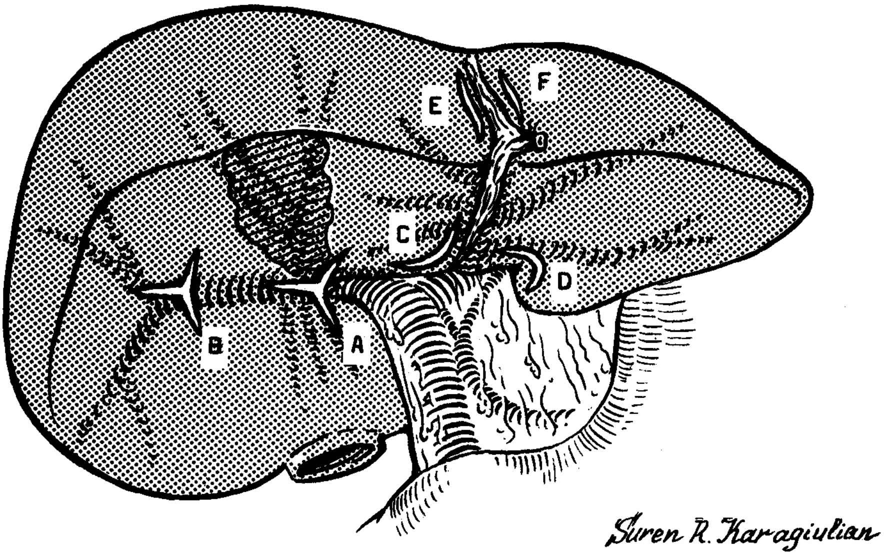

**Figure 1** External landmarks and sites for incising Glisson's capsule on the undersurface of the liver and approaching lobar and segmental pedicles.
A — for pedicle of right lobe, SV and SVIII
B — for pedicle of right lateral lobe (SVI, SVII) and segmental pedicles of SVI and SVII
CD — for left lobar pedicle (SII, SIII, SIV)
EC — for pedicle of SIV
FD — for pedicles of SII and SIII

* Classification after C. Couinaud.[2]

with that of the intended resection. In topographic studies the extent of ischemia was imitated by perfusing the hepatic vessels with a contrast medium after compressing the hepatic pedicle.

Thus, the entire manipulation of transhepatic finger exposure of pedicles (FEP) consists of four successive stages: 1) a superficial incision of Glisson's capsule at a point related to external landmarks, 2) fingertip intraparenchymatous exploration for a large trunk — internal landmark, 3) hooking the HP and drawing it out onto the visceral surface of the liver, 4) correcting the position of the clamp preliminarily placed on the HP according to the borderline of liver ischemia.

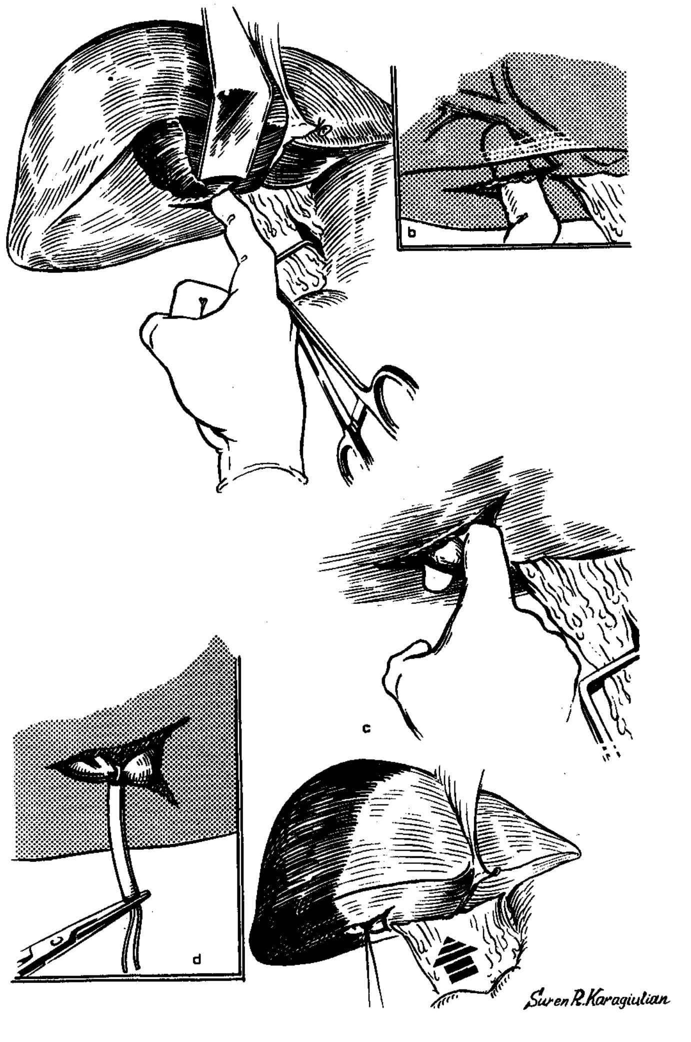

**Figure 2** Stages of transhepatic finger exposure of a pedicle a to e — steps of procedure (see text).

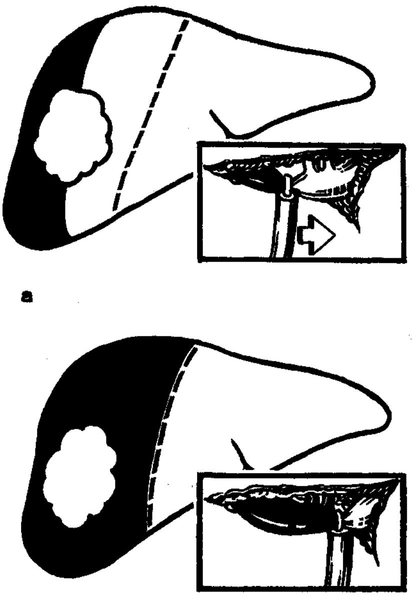

a b

**Figure 3** Selective control of liver ischemia
a — the borderline of ischemia fails to coincide with the intended line of resection (interrupted line): shift the clamp proximally to involve additional branches;
b — correct placement of clamp: may be replaced by ligature.

This method of selective FEP has been used in eight patients aged between 15 and 51 years when performing right hepatectomy (5), right lateral lobectomy (2) and left caval lobectomy (1).

### RESULTS

Anatomic studies allowed to determine more accurately the depth of lobar and segmental HP lodgement from the visceral surface of the liver (Table 1), and to detect the external and internal landmarks for HE of various scope and the direction for finger tunneling of the liver parenchyma.

The deepest HP lodgement was observed for the Vth and VIIIth segments while HP lodgement nearest the surface was in the IInd and IIIrd segments.

The elasticity of the hepatic pedicles enfolded in an epifascial sheath was adequate for being hooked by the finger and drawn out through the slit on the liver's undersurface irrespective of the depth of HP lodgement.

The external and internal landmarks for the detection of lobar and segmental HPs are presented in Table 2 and also in Figure 1.

Given below are the specific features of performing hepatectomies of various scope with the use of selective FEP.

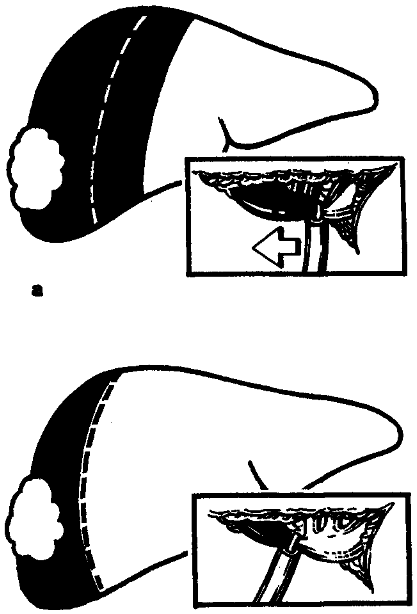

a b

**Figure 4** Selective control of liver ischemia
a — ischemia exceeds intended scope of resection (interrupted line): move clamp distally, releasing 1 or 2 of the grasped pedicle's lateral branches.
b — correct placement of clamp: may be replaced by ligature.

**Table 1** Depth of lobar and segmental HP lodgement from the liver's undersurface

| *HP of* | *Depth of lodgement, cm* | | |
|---|---|---|---|
| | *Max* | *Min* | *M ± m* |
| Right lobe | 1.8 | 0.8 | 1.4 ± 0.1 |
| Left lobe | 1.5 | 0.6 | 1.1 ± 0.1 |
| Segmental: | | | |
| SII | 1.7 | 0.8 | 1.3 ±0.2 |
| SIII | 1.9 | 1.3 | 1.7 ± 0.1 |
| SIV | 2.5 | 1.2 | 1.8 ± 0.1 |
| SV | 3.0 | 1.8 | 2.2 ± 0.2 |
| SVI | 2.1 | 0.7 | 1.4 ± 0.1 |
| SVII | 2.5 | 1.6 | 2.2 ± 0.1 |
| SVIII | 4.0 | 2.9 | 3.8 ± 0.2 |

*Right hepatectomy* (removal of segments V, VI, VII, VIII). The operation's main stage is the exposure and ligation of the right lobar HP. After performing a cholecystectomy a superficial T-shaped incision is made on the liver capsule at the point where the posterior edge of the gallbladder fossa touches the right part of the porta hepatis (see Figure 1.A). Having clamped the HDL, and slightly

**Table 2** External and internal landmarks for detecting lobar and segmental HP

| *Nos. HP of* | *External landmarks* gallbladder fossa | *porta hepatis* | *round ligament* | *Internal landmarks* | *Designations in Fig. 1* |
|---|---|---|---|---|---|
| 1. Right lobe (SV, SVI, SVII, SVIII) | posterior edge | right part | | right lobar HP | A |
| 2. Right lateral lobe (SVI, SVII) | right edge | mental extension to the right | | HP of SVI | B |
| 3. Right para-median lobe (SVI, SVIII) | posterior edge | | | 1st large branch of right HP | A |
| 4. Left lobe (SII, SIII, SIV) | | left part | inferior-medial edge | left HP | CD |
| 5. Left caval lobe | | left part | lateral edge | HP of SIII | FD |
| 6. IVth segment | | central part | super-medial edge | left HP | CE |

stretching it by downward traction, the surgeon, glides his forefinger along its right edge and, continuing in this direction, introduces the forefinger through the slit into the parenchyma and further slides it along the epifascial sheath of the right lobar HP. Bending his forefinger around this HP anteroposteriorly, the surgeon detaches it from the surrounding parenchyma after which he pulls it out through the slit by gentle traction downwards and places on it a provisional clamp or tourniquet while relieving the HDL, thereby restoring circulation in the liver parts to be retained.

In topographical and anatomic studies the duration of HDL compression, sufficient for "bloodless" exposure of the right lobar HP, did not exceed 2 min. In 4 liver preparations out of 10 the portal branches of the Ist segment rose in a bunch from the beginning of the right lobar portal pedicle, which created certain difficulties in exposing this HP since its grasping and withdrawal at the level of or proximal to these branches, may result in tearing them or putting part of SI out of circulation. In such cases the lobar HP should not be immediately hooked by the finger but the latter should glide along its longitudinal axis while going in spiral-fashion around it anteroposteriorly.

By perfusing the portal branches with a contrast medium it was established that in none of the 10 observations did the ligature entangle branches leading to other anatomical portions of the liver not intended for removal.

*Right lateral lobectomy* (removal of segments VI and VII). The main stage is the exposure and ligature of the right lateral pedicle proximal to its division into HP of SVI and SVII. The outward landmark for slitting the capsule is the point of crossing of two hypothetical lines: a line parallel to and 2 cm to the right of the gallbladder fossa, and the line of the porta hepatis extended to the right (see Figure 1 B). The internal landmark is HP of SVI which, as a rule, is well pronounced. After making a superficial T-shaped incision of Glisson's capsule at this point the forefinger should be introduced to the depth of 2–2.5 cm — deeper than for detecting the right lobar HP.

In eight observations out of 10 simultaneously withdrawn through the incision were the HP of SVI and VII, in one — the area of bifurcation and in one — the area of bifurcation and in one — the HP of SVI. In nine observations the ligature captured the trunks of the right lateral lobe alone, in one the contrast medium during perfusion did not penetrate the right portions of SV. The ligature was moved to the right, relieving the 1st lateral branch and upon repeated perfusion all the portions of SV were adequately filled. Shifting the ligature did not require additional HDL clamping and the duration of general liver ischemia was determined by the time required for the detection and withdrawal of the HP, coming to 1.5 min.

*Right paramedian lobectomy* (removal of segments V and VIII). The main stage is the exposure and ligation of the common HP of SV and SVIII. The approach was the same as for right hepatectomy (see Figure 1 A); however, after introducing the forefinger into the incision of the capsule, its tip tunneling the parenchyma slips along the anterosuperior surface of the right lobar HP, which serves in this case as a reliable internal landmark until it comes against the first large branch (sometimes there are 2–3 or more of them) which is the HP of the paramedian lobe. This is the HP that should be ligated as far away as possible from the right lobar HP lest it interferes with the circulation of SVI and VII. The duration of HDL clamping did not exceed 2 min. However it must be noted that when capturing a rather thin (2–3 mm) HP a second attempt should be made 0.5–1 cm deeper where, in case of the scattered type of these segments' circulation, 2–3 additional branches are situated, or the short wide HP (0.5–0.8 cm ) of SV and VIII.

*Left hepatectomy* (removal of segments II, III and IV). The main stage is the exposure and ligation of the left lobar HP. The external landmark for slitting the capsule is the point where the median edge of the fissure of the liver's round ligament crosses the central portion of the porta hepatis (see Figure 1 C); the specific position of this HP requires an additional incision at the left end of the porta hepatis (see Figure 1 D). The fingertip introduced into incision C, this time no deeper than 1–1.5 cm, comes across a large trunk which is the left lobar HP. The finger should be advanced spiral-fashion anteroposteriorly and somewhat to the left towards incision D, making a tunnel over the lobar HP which is then drawn outside with part of the porta hepatis, joining both capsule incisions into a single one (Figure 5 a, b). In one case the fingertip came across a large branch rising from the posterosuperior surface of the left HP which we mistook for the branch going to SII and bypassed it on the median side. However, during perfusion with a contrast medium it failed to enter the right paramedian lobe, thus enabling us to diagnose a variant of an SV and SVIII HP rising from the left lobar HP (at any rate of its portal vein through which the perfusion was performed), confirmed by subsequent dissection of the HP elements after dissecting its epifascial sheath. In nine other preparations the limits of the "ischemia" corresponded to those of the left lobe. Duration of HDL clamping was 1.5–2 min.

*Left caval lobectomy* (removal of segments II and III). The main stage is the exposure and ligation of the HP of SII and III. The external landmark is the point where the lateral edge of the round ligament crosses the porta hepatis (see Figure 1 D). The internal landmark for the fingertip is the well pronounced HP of SIII, moving along which one may detect the bifurcation of the portal pedicles of the left lobe. However, in three preparations the distance between the pedicular stoma of SII and SIII exceeded 2.5 cm and in order to avoid rough ruptures of the parenchyma they were ligated separately, upon adding the incision F (see Figure 1 F): first ligated was the HP of SIII and then of SII. Subsequent perfusion demonstrated that in all

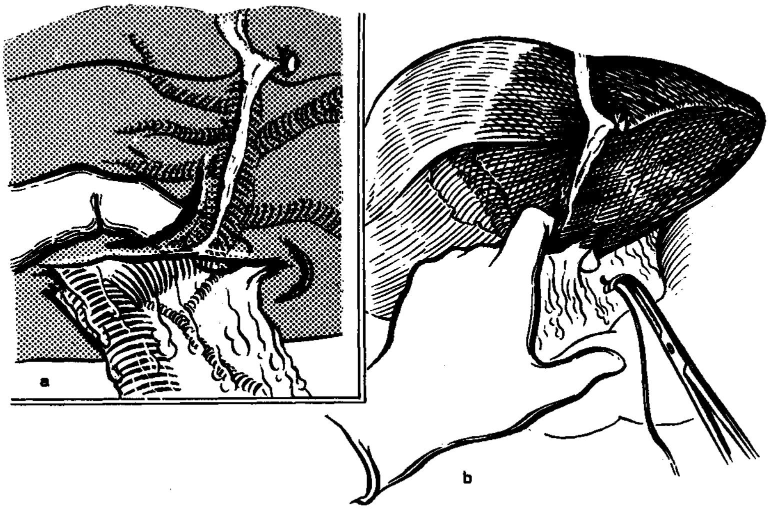

**Figure 5** Finger exposure of left lobar pedicle a and b — steps of procedure (see text).

10 liver preparations, the placement of ligatures corresponded to the aferent branches of the left caval lobe. Duration of HDL clamping did not exceed 2.5 min.

*Segmentectomy, SIV.* Isolated resection of segment IV was elaborated first of all for purposes of right extended hepatectomy in which one of the stages includes the removal of this segment. The main difficulty here is the exposure and ligation of all the vascular branches in SIV without interfering with the circulation of SI and the left caval lobe. The external landmark for the slit is the point where the medial edge of the round ligament crosses the porta hepatis (see Figure 1 C). This is a wide incision upwards along the round ligament, then along the diaphragmal surface along the falciform ligament (or a separate additional slit of the capsule to the right of the falciform ligament — see Figure 1 E). The internal landmark is the left lobar HP. The free end of the round ligament is seized with a clamp and reverted upwards and to the left. The surgeon introduces his forefinger into the upper slit near the falciform ligament and glides it downwards along the medial surface of the left lobar HP (practically along the surface of the portal sinus of Rex) towards the lower incision near the porta hepatis (Figure 6). During this maneuver a parenchymatous bridge with HP branches of SIV passing within it occurs between the introduced forefinger and the opposing thumb outside. The parenchyma is crushed by massaging movements of the thumb and the several HP branches of SIV, hooked by the finger, are brought out through the rupture along the round ligament to be ligated one after another. Here the duration of HDL occlusion did not exceed 3 min. In only one of 10 observations the preliminarily placed ligature captured an atypically situated branch which supplied the SIII, a fact established during perfusion with contrast medium and confirmed by subsequent dissection.

The elaborated method of selective FEP was used in 8 patients, for whose basic data see Table 3.

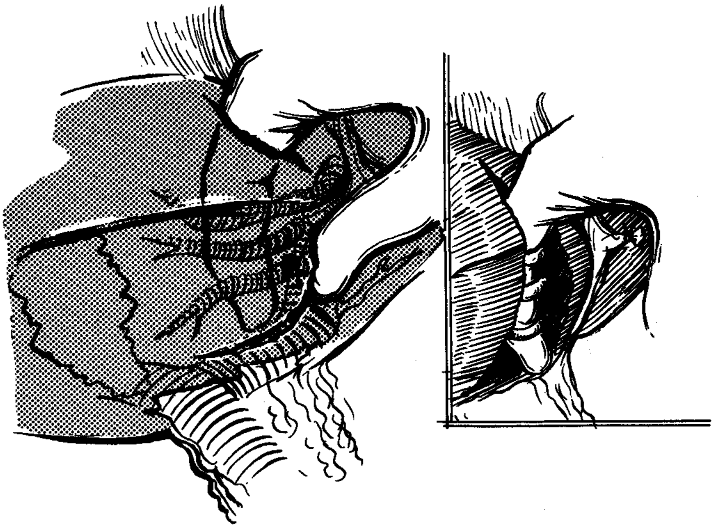

**Figure 6** Finger exposure of pedicle of IVth segment (see text).

**Table 3** Basic information on patients who underwent HE by the method of selective FEP

| *Nos.* | *Sex* | *Age (years)* | *Diagnosis* | *Scope of surgery* | *Loss of blood, ml* | |
|---|---|---|---|---|---|---|
| | | | | | *upon HP exposure* | *total* |
| 1. | f | 24 | Mesenchymal hamartoma of right lobe, external pyobiliary fistula | Right hepatectomy | 200 | 700 |
| 2. | m | 48 | Cavernous hemangioma | Right hepatectomy | 300 | 800 |
| 3. | f | 51 | Echinococcosis | Left caval lobectomy | 150 | 200 |
| 4. | f | 15 | Liver cell carcinoma | Right hepatectomy | 250 | 1100 |
| 5. | f | 44 | Chronic abscess | Right lateral lobectomy | 250 | 1500 |
| 6. | f | 47 | Cavernous hemangioma | Right lateral lobectomy | 300 | 1000 |
| 7. | f | 41 | Hemangioma | Right hepatectomy | 150 | 700 |
| 8. | f | 36 | Suppurated non-parasitogenic cyst | Right hepatectomy | 200 | 2000 |
| | | | | $M \pm \sigma$ | 225±59.8 | 1000±550 |

Clinical case history: a 48-year-old patient was admitted to our clinical department on January 5, 1984, complaining of evening rise of temperature to 38–39°C, chills. In 1977 enlargement of the liver was detected for the first time in an outpatient setting which, however, was not accompanied by any subjective sensations and was detected quite accidentally. Since December 1983, the patient began feeling heaviness in the right hypochondrium to which elevated temperature was soon added. Underwent examination at a surgical hospital at the place of residence: radioisotope scanning revealed some formation in the right lobe of the liver; selective celiography showed an angiographic picture characteristic of hemangioma of the liver; this diagnosis was confirmed by laparoscopy, yet biopsy material contained no tumoral cells.

General status on admission was satisfactory. The liver protrudes by 8 cm and has a dense-elastic consistency. X-raying showed elevation of the right diaphragmatic dome. Negative reaction to alpha-phetoprotein, reactions with echinococcal antigen also negative.

Surgery on January 27, 1984 — right hepatectomy performed from a bilateral subcostal incision. The area of SV, VI, VII, VIII revealed a tuberose red-violet tumor of softly elastic consistency, 23 × 17 × 11 cm in size. Left lobe compensatorily hypertrophied. Patent portal and caval porta. Tumor is fused with right diaphragmatic dome. The right lobe was mobilized by dissecting ligaments and adhesions to the diaphragm. Gallbladder was removed. The hepatoduodenal ligament was compressed by a tourniquet and the right lobar HP was exposed by the method of selective FEP within 3 min. Total blood loss at this stage was less then 300 ml. Further resection was performed by digitoclasia along the ischemic borderline. Total intraoperative blood loss 800 ml. Histologically supported conclusion: multiple cavernous hemangiomas with necrotic foci. Discharged on 27th day. Four years later healthy and employed in his principal occupation.

### DISCUSSION

The number of hepatectomies performed around the world and of publications about them is progressively mounting. According to J. Fortner,[9] from 5000 to 6000 new candidates for extensive HE appear every year in the USA alone. Real possibilities, however, are much more modest. Most authors perform from 100 to 150 HE, seldom more, over 10 to 20-year periods.

As the same time a hepatectomy remains the operation of election in the majority of tumoral lesions of the liver.[17] Hence quite understandable is the striving to make this operation less traumatic and better available to a greater number of surgeons. Recent years have seen considerable strides in this direction. In this two main tendencies can be clearly seen: prolongation of complete occlusion of general hepatic circulation during the most traumatic stages of the operation to 30–60 min of normothermic ischemia and even longer (up to 90–120 min) with local external cooling,[4,8,14] and also the use of new instruments based on modern physical principles ensuring bloodless separation of parenchyma and the exposure from it of pedicles for isolated ligation: various modifications of lasers,[21] an ultrasonic scalpel,[16,20] a plasma[11] and cryogenic[1] scalpel, a high-pressure water jet,[15] and some other devices.

The possibility of maintaining normothermic ischemia of the liver for longer than was formerly conceived, became apparent as a result of accumulated clinical experience which demonstrated that, in distinction from dogs (which actually tolerate ischemia for no longer than 15–20 min), quite important in man are the natural portocaval shunts which prevent blood engorgement of the abdominal viscera.[4] At the same time this advantage is probably considerably restricted during extensive resections of a liver altered by cirrhosis, chronic hepatitis and fibrosis, when the tumoral process is widespread, during liver trauma involving heavy loss of blood, i.e. in states when the amount of retained functional hepatic tissue is relatively small and its lasting ischemia may cause the development of postoperative hepatic insufficiency (HI).

Thus, E. Delva *et al.*[4,5] report a substantial diminution of intraoperative blood loss in 90 per cent of cases on account of liver ischemia lasting up to 45–90 min. However, postoperative HI developed in 10 patients, in 6 of them the liver being diffusely altered. Similar data come from other authors as well: HI developed in five patients in the early and in 11 in the later postoperative period out of 130 patients who underwent extensive HE against a background of chronic concurrent liver diseases.[22] In recent years many authors[1,10,14,18] regard HI to be the main liver resection problem associated with the overestimation of the functional capacities of what remains from the organ. Their published results make one pick one's steps and testify that lasting ischemica of the entire liver seldom leaves no consequences and the matter, therefore, calls for reconsideration.

The experience of recent years indicates that diminishing intraoperative blood loss by using more perfect instruments seems much more promising, even though they are at times rather sophisticated and require special skills.[16]

Our method by no means excludes the use of such instruments for bloodless HP exposure; it even simplifies the operation, since temporary ligation or clamping of the exposed HP immediately and clearly shows the true extent to which the operation's basic stage may be safely carried out by using a parenchyma ultrasonic aspirator or some other "bloodless" surgical instrument strictly along the line of demarcation, i.e. by completely observing the principle of anatomic resection without the hazard of accidental ligation of trunks supplying the organ's territory not intended for removal. The method is simple enough and generally accessible since it requires no complicated geometric computations for accurately spotting the site of HP projection as it utilizes the principle of internal fingertip orientation and adjustment of the ligature or clamp by shifting it along the exposed HP, nor does it require additional three-dimensional angiographic examinations (which is especially important in an urgent situation), for even when atypical departures of a lobar artery or a portal vein branch are encountered, the branches supplying the segment or sector to be removed are situated precisely in the common epifascial sheath of the HP corresponding to the territory concerned.

### *References*

1. Alperovich, B.I. (1983) *Liver Surgery*, Tomsk (in Russian).
2. Couinaud, C. (1957) Le foie. Etudes anatomiques et chirurgicales. Paris: Masson.
3. Couinaud, C. (1981) Controlled hepatectomies and exposure of the intrahepatic bile ducts. Anatomical and technical study. Paris.
4. Delva, E., Camus, Y., Lienhart, A. *et al.* (1986) Hemodynamic effects of portal triad clamping in man. *Abstr. 1st World Congr. HPB Surgery*, Lund, Sweden, June 9–13th, p. 346.
5. Delva, E., Parc, R., Hannoun, L. *et al.* Experience with 153 liver resections during the last 6 years. *Abstr. 1st World Congr. HPB Surgery,* Lund, Sweden, June 9–13th 1986, p. 75.

6. Galperin, E.I. and Mochalov, A.M. (1986) Transhepatic finger exposure of lobar and segmental hepatic pedicles for the anatomical resections of the liver. In *Khirurgiya*, No. 7 pp. 3–9 (in Russian).
7. Galperin, E.I., Karagiulian, S.R. and Mochalov, A.M. (1987) Experience in anatomical and atypical hepatectomies. In *Khirurgiya*, No. 7, pp. 56–62 (in Russian).
8. Esser, C. Liver surgery with continuous interruption of blood circulation. *Abstr. 1st World Congr. HPB Surgery*, Lund, Sweden, June 9–13th 1986, p. 326.
9. Fortner, J.G., and Silva, J.S., Golbey, R.B. *et al.* (1984) Multi-variate analysis of a personal series of 247 consecutive patients with liver metastases from colorectal cancer. I. Treatment by hepatic resection. *Ann. Surg.*, Vol. 199, No. 3, pp. 306–316.
10. Kremer, B. and Bruns, D.H. 139 liver-resections: results and problems after extensive resections. *Abstr. 1st World Congr. HPB Surgery*, Lund, Sweden, June 9–13th 1986. p. 94.
11. Link, W.J., Incropera, F.P. and Glover, J.L. (1976) A plasma scalpel. Comparison of tissue damage and wound healing with electrosurgical and steel scalpels. *Arch. Surg.*, Vol. 111, pp. 392–397.
12. Nordlinger, B., Douvin, D., Javaudin, L. *et al.* (1980) An experimental study of survival after two hours of normothermic hepatic ischemia. In *Surgery Gynecol. Obstet.*, Vol. 150, pp. 859–864.
13. Ostroverkhov, G.E., Zabrodskaya, V.D. and Zatolokin, V.D. (1968) Anatomical substantiation of lobar resections of the liver. Communication 1. In *Khirurgiya*, No. 5, p. 151 (in Russian).
14. Pachter, H.L., Spencer, F.C., Hofstetter, S.R. *et al.* (1983) Experience with the finger fracture technique to achieve intrahepatic hemostasis in 75 patients with severe injuries of the liver. In *Ann. Surg.*, Vol. 197, No. 6, pp. 771–778.
15. Papachristou, D.N. and Barters, R. (1982) Resection of the liver with a water jet. *Brit. J. Surg.*, Vol. 69, No. 2, pp. 93–94.
16. Putnam, C.W. Hepatic resections facilitated by ultrasonic dissection. *Abstr. 1st World Congr. HPB Surgery*, Lund, Sweden, June 9–13th 1986, p. 325.
17. Ringe, B., Neuhaus, P., Bechstein, W.O. *et al.* Experience with liver resection and transplantation for hepatocellular carcinoma. *Abstr. 1st World Congr. HPB Surgery*, Lund, Sweden, June 9–13th 1986, p. 373.
18. Saito, H., Makishima, T., Nagao T. *et al.* Multiple organ failure as a fatal complication of hepatectomy. *Abstr. 1st World Congr. HPB Surgery*, Lund, Sweden, June 9–13th 1986, p. 223.
19. Schapkin, V.S. (1967) Hepatic resection. Moscow: Meditsina.
20. Scheele, J., Richter, H. and Altendorf, A., Ultrasonic aspirator and fibrin tissue adhesive in liver resection. *Abstr. 1st World Congr. HPB Surgery*. Lund, Sweden, June 9–13th 1986, p. 48.
21. Skobelkin, O.K., Litvin, G.D. and Yeliseyenko, E.I. (1982) Experimental and clinical substantiation of the use of various sources of laser emission in liver surgery. In *Physiology and Surgery of the Liver*. Tomsk, pp. 102–104 (in Russian).
22. Tung, T.T. (1962) Chirurgie d'exerese due foie. Hanoi.
23. Yakaya, H., Nagasue, N., Ogawa, Y. *et al.* Clinical experience with 132 hepatic resections for hepatocellular carcinoma. *Abstr. 1st World Congr. HPB Surgery*. Lund, Sweden, June 9–13th 1986, p. 88.

*Accepted by L. Blumgart on 28 July 1988.*

